# Undergraduate medical research in the Gulf Cooperation Council (GCC) countries: a descriptive study of the students’ perspective

**DOI:** 10.1186/s13104-018-3381-y

**Published:** 2018-05-08

**Authors:** Zaid Sayedalamin, Taher Fawzy Halawa, Mukhtiar Baig, Osama Almutairi, Hassan Allam, Tahir Jameel, Zohair Jamil Gazzaz, Hazem Atta

**Affiliations:** 1 Department of Surgery, Faculty of Medicine in Rabigh, King Abdulaziz University, Jeddah, Saudi Arabia; 2 Department of Pediatrics/Medical Education, Faculty of Medicine in Rabigh, King Abdulaziz University, Jeddah, Saudi Arabia; 3 Department of Clinical Biochemistry/Medical Education, Faculty of Medicine in Rabigh, King Abdulaziz University, Jeddah, Saudi Arabia; 4 Department of Community Medicine, Faculty of Medicine in Rabigh, King Abdulaziz University, Jeddah, Saudi Arabia; 5 Department of Obstetrics and Gynecology, Faculty of Medicine in Rabigh, King Abdulaziz University, Jeddah, Saudi Arabia; 6 Department of Medicine, Faculty of Medicine in Rabigh, King Abdulaziz University, Jeddah, Saudi Arabia; 7 Department of Clinical Biochemistry, Faculty of Medicine in Rabigh, King Abdulaziz University, Jeddah, Saudi Arabia; 8 Department of Medical Biochemistry and Molecular Biology, Faculty of Medicine, Cairo University, Cairo, Egypt

**Keywords:** GCC, Undergraduate medical research, Research attitude, Research barrier

## Abstract

**Objective:**

There is a lack of research-oriented physicians in several Arab countries and especially in Gulf region countries. In this context, it is important to explore medical students’ perceptions and motivations towards research. The aim of the present study was to investigate research attitude, practices, and motivations among medical students from GCC countries.

**Results:**

There were 228 students who participated in this study (male 88, females 140). Thirty-eight percent of the students were participating from Saudi Arabia, 20.6% from the UAE, 17.1% from Oman, 12.7% from Kuwait and 11.4% from Bahrain. Among participants, 43.0% had experience of funded research, and 53.1% had a contribution to research. The confidence of participants in their ability to interpret and to write a research paper was quite high (70.2%). The majority of the students (87.3%) believed that undergraduate students could conduct research and can present at conferences. Improving research skills, attaining research publication, and improvement in patient care were claimed as the top three motives for conducting research. The majority (75.0%) were compelled to research to facilitate their acceptance to a residency program and 63.6% due to compulsion for a research methodology course.

**Electronic supplementary material:**

The online version of this article (10.1186/s13104-018-3381-y) contains supplementary material, which is available to authorized users.

## Introduction

Medical research is of tremendous benefit in medical education. The newer trends of teaching in medical education focus mainly on acquiring the essential skills of the students rather than learning by heart [1]. Teaching activities like Problem-Based Learning (PBL) contribute towards targeted literature search by the students. This habit then leads to positive research activities [2]. Now it is widely acknowledged that, if medical education is successful in developing creative thinking, it forms a basis of research-oriented and creative physician-scientists of the future [3]. There is sufficient evidence that involvement in research activities in early medical training leads to diverse postgraduate research inclinations in professional career [3, 4].

A study reported that practicing physicians do not have sufficient awareness regarding related medical databases, medical journals, and review publications [5]. Therefore, research at the undergraduate level will improve their awareness of medical databases and medical literature. Additionally, it will help in acquiring the art of critical thinking and a creative attitude towards management of locally prevalent diseases. The students who are exposed to research activities in their graduate years are more likely to follow principles of evidence-based medicine and make rational decisions in difficult clinical situations [6]. The presence of research-oriented atmosphere by the medical school faculty results in the observation of positive research trends in undergraduate medical students [7].

Generally, one observes that most of the research is being carried out in the Western countries and comparatively very little organized research is visible in Asian and Middle Eastern countries. Most of the doctors prefer being a clinically-oriented physician and their inclination towards research is minimal [8]. Exposing students to research activities is the only way to inculcate the habit of research in young doctors [9]. The aim of our study is to assess the research attitude, practice and motivation among the students from GCC so that the corrective and adaptive measures are implemented for the research-promoting environment in GCC region medical schools.

## Main text

### Methods

#### Study design

The present cross-sectional study was carried out during the Ninth International Scientific Conference for Medical Students in the GCC Countries in Alain, United Arab Emirates (UAE), in December 2014.

#### Study participants

In the present study, only those medical students and interns who attended the Ninth International Scientific Conference for Medical Students in the GCC Countries in Alain, UAE, were included. The questionnaire was developed with the help of previously published studies [10, 11] with some modifications and additions (attached as Additional file 1). The questionnaire was pretested and verified for errors on a group of 50 students and modified accordingly. The reliability of the questionnaire was determined by measuring the related Cronbach’s Alpha, which was equal to 82%, indicating good consistency in the responses from study participants. A self-reported questionnaire was administered to medical students who attended the conference. There were around 700 registered students who attended the conference, but several students came from Canada, USA, Poland, Britain, Yemen, and Sudan. Actually, our college is a comparatively new medical college located 150 Kilometers away from Jeddah city [12]. Therefore, our college established a special booth in that conference to introduce our college, and we invited all the students who visited our booth. We only included those students who were studying in the GCC. Out of approximately 500 students studying in GCC, 262 (response rate 52%) students’ gave their consent and participated. However, only 228 questionnaires were completely filled, so we include only 228 participants. Our questionnaire consisted of several parts; the first part was related to demographic data, and in the second part, there were few questions related to the personal experience of participants in research and their beliefs based on that experience. The third part contained 3-point Likert scale questions about their motive behind conducting research during medical school and their perception of the barriers against students’ research. The fourth part related to the type of studies conducted by those who have participated in medical research earlier.

#### Statistical analysis

Data was analyzed on SPSS-20. The qualitative variables were represented by the frequency and percentages. Academic year-wise comparison of students and interns regarding motives of research and research barriers was done by *χ*^2^ test. The p value  < 0.5 was considered significant.

### Results

There were 228 students who participated in this study; among them 88 (38.6%) were males, and 140 (61.4%) were females. Most of the students 87 (38.2%) were participating from Saudi Arabia, 47 (20.6%) from the UAE, 39 (17.1%) from Oman, 29 (12.7%) from Kuwait and 26 (11.4%) from Bahrain. Most of the students participating in the conference were of third or later year students, but there were 5 (2.2%) first-year medical students and 18 (7.9%) second-year students.

There were 108 (47.4%) who were presenting their research along with 27 (11.8%) as coauthors. There were 10 (4.4%) organizers, and the remaining 83 (36.4%) were only attending the conference as the audience (not shown in the table).

Among participants, 98 (43.0%) had experience of funded research, and 121 (53.1%) had a contribution in research, in a topic related to the social impact of GCC. The confidence of participants in their ability to interpret and write a research paper was quite high (70.2%), and they believe that undergraduate students can conduct research and present even higher (87.3%), but still there were only 71 (31.1%) who believed they could do it without supervision (attached as Additional file 2: Table S1).

Improving research skills, attaining research publication and improvement in patient care were claimed as the top three motives for conducting research. It was important to note that the majority (75.0%) were compelled to research to facilitate their acceptance to a residency program and 63.6% due to compulsion for a research methodology (RM) course. There was no significant difference between various years’ students in agreeing for motives of research (attached as Additional file 3: Fig. S1; Additional file 4: Table S2).

**Fig. 1:**
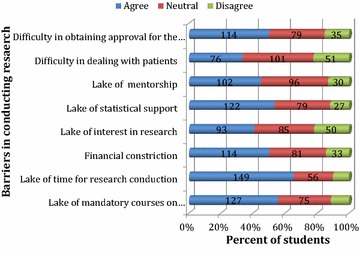
Agreement status of students for considering barriers while conducting research

Lack of time was claimed to be the most prominent barrier to 65.4% students, followed by the lack of mandatory courses on RM, statistical support, financial constraints, and approval process of research by 55.7, 53.5, 50 and 50%, respectively. Lack of mentorship and difficulty in dealing patients were claimed to be the barriers to conducting research by 44.7% and 33.3%, respectively (Table 1; Fig. 1).

**Table 1:** Academic year-wise comparison of students regarding barrier for conducting research

Barriers	Academic year							p value
	1st year	2nd year	3rd year	4th year	5th year	6th year	Intern	
Lack of mandatory courses on research methodology	40.0	38.9	43.5	61.1	58.5	61.5	73.7	0.25
Lack of time for research conduction	60.0	61.1	60.9	61.1	73.8	66.7	57.9	0.46
Financial constriction	40.0	44.4	39.1	61.1	50.8	51.3	57.9	0.52
Lack of interest in research	40.0	33.3	26.1	47.2	44.6	43.6	52.6	0.65
Lack of statistical support	20.0	44.4	52.2	55.6	44.6	64.1	78.9	0.11
Lack of mentorship	40.0	33.3	43.5	47.2	41.5	46.2	63.2	0.42
Difficulty in dealing with patients	60.0	16.7	45.7	27.8	33.8	28.2	31.6	0.33
Difficulty in obtaining approval for the study	80.0	50.0	58.7	41.7	46.2	51.3	47.4	0.17

Results are shown as percentage (%)

**Fig. 2:**
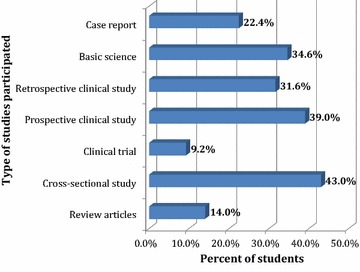
Participation of students in research by various types of research

There were 107 (47%) students who had participated earlier in different types of research activities in their college (Fig. 2).

### Discussion

Awareness of the positive effects of medical research on medical education itself and patient care is strongly associated with the involvement of medical students in research activities. It is well established that the medical students of GCC are now well aware of the importance of undergraduate research activities and this trend is because of significantly raised understanding of the research training being taught in medical schools and universities [13–15]. The last few years have witnessed improved curriculum with more emphasis on research motivation and acknowledgment of the students with better options for residency and postgraduate placements [16].

Our study was unique in the sense that our participants represented the whole GCC, i.e., Saudi Arabia, UAE, Oman, Kuwait & Bahrain. For quality control, the questionnaire for our study was pretested and verified for any error on a group of 50 random medical students.

In contrast to several other studies carried out in our region, the majority of our participants were female students (61.4%) [13, 17, 18]. As most of our participants were from senior classes (80%), we had a chance to inquire from the cohort that was already well aware of requirements and demands of executing undergraduate research projects. Around 60% of students/interns were either themselves presenting their research or were the co-authors. A study revealed that students can be extraordinarily active in research projects provided suitable environment and encouragement is ensured [19].

Around 30% were confident of their abilities to conduct a feasible research project in case of availability of adequate mentorship. When asked specifically 33.3% of the participants revealed the difficulty in starting any research project in the absence of mentorship. Studies concerning the trend of medical undergraduate research in well-established medical institutes where professional mentorship is readily available show a similar trend in students [20, 21].

The majority of participants mentioned that the curriculum in their respective medical university was arranged in such a way that around 63.6% of them presented the research work to complete their research methodology course. Khan et al., in 2015, showed that changes in curriculum and the addition of a research methodology course totally ended the hesitation towards research among medical students [22]. The majority of them had the goal of improving their research skills and attaining research publications to get a proper residency program. This concern is so strong among the medical students that even in very junior classes 80% of students mentioned it as a strong motive [23].

Some other motives such as attaining a research publication in a student’s life were seen to be in the range of 80% to the first year medical student to almost 78.9% during the internship. All the groups were almost equally interested as is evident by its p value of 0.73. Two recent international studies revealed a similar trend in their students [13, 24].

Regarding the various barriers in initiating and working on a research project, there were many obstacles for our participants and the severity of various barriers varied widely with the seniority (from first-year medical student to the interns) of participants, and a similar trend has been mentioned in the literature [25]. Lack of mandatory courses, a pinch that was felt by all of the participants in varying degrees and the highest number of Interns complained mostly about it. Mandatory courses for research methodology are part of the curriculum for the last few years only and seniors seem justified, as they did not have a chance to learn the essential methodology [26].

Almost all the participants to varying degree irrespective of their seniority mentioned the lack of time to participate in research activities. The role of mentorship is very important in this respect as undergraduate research activities can be managed parallel to the curricular commitments provided students are guided by a professional facilitator [27]. Research is generally taken as a full time endeavor, and when the students are busy in their academic activities there is a general trend of lack of interest in research commitments [28], and a similar trend was seen in our study.

Another very important barrier in research activities is the approval by the institutional ethical committee. Most of the time ethical committee documentation is very exhaustive, and a lot of objections distract the young researchers [29]. But our survey revealed that with the advance in seniority of the students, when the young researchers get to understand the procedure better, this barrier occurs less, i.e. it fall from 80% among the first year students to 47.4% in interns.

We suggest that the student’s motivation and conducive research environment is of tremendous significance in generating undergraduate research activities among medical students and young doctors. We further suggest that the regulatory and registering councils in GCC countries should lay emphasis on students’ research and make it mandatory for new doctors’ registration or give a few extra points for their published research paper and participation in research as the Saudi Council for Health Specialties has recently done.

### Conclusion

The majority of the participants in our study revealed their commendable interest in research activities and made valuable contributions to almost all the research fields. However, the respective countries’ universities should also encourage the faculties to supervise and guide the students’ research projects, and universities should provide sufficient funding to students’ research projects. Further studies are recommended with larger sample sizes to confirm our results.

## Limitations

This is a questionnaire-based study. Therefore, the participants’ biases cannot be ignored, and there was no method to check the correctness of participants’ responses. One important limitation is the sampling of our research participants as they are attending a scientific conference and many of them were making presentation, so one can assume that they are more likely to be the ones interested in research. Moreover, our sample size was small, so these results cannot be generalized.

## Additional files

**Additional file 1.** Study questionnaire.

**Additional file 2: Table S1.** Personal experience of participants in research and their believes based on that experience.

**Additional file 3: Figure S1.** Agreement status of students with motives of conducting research.

**Additional file 4: Table S2**. Academic year-wise comparison of students regarding motives of research.

## Abbreviation

GCC: Gulf Cooperation Council
