# Supplementary material for: Undergraduate medical research in the Gulf Cooperation Council (GCC) countries: a descriptive study of the students’ perspective
Source: BMC Res Notes. 2018 May 8;11:283. doi: 10.1186/s13104-018-3381-y (PMC5941694; doi:10.1186/s13104-018-3381-y)
Supplement: Supplementary file 1 — Additional file 1. Study questionnaire. [file 13104_2018_3381_MOESM1_ESM.docx]

**Questionnaire**

**Age:** (yrs) ------ **Gender:** Male/Female **Academic year:** 1^st^/2^nd^/3^rd^ /4^th^ /5^th^ 6^th^ /Intern

**College/University name:**

**Country name:**

**You are attending this conference as:**

1. presenter b) coauthor c) just attending d) other

**Mode of learning in your school:**

a) Case based learning b) Problem Based Learning (PBL) c) Team Based Learning (TBL)

d) Traditional e) All of the above f) Other

**What is your own method for preparation to pass the exams?**

1. Q-bank b) Lectures c) Textbooks d) Other

**Have you ever presented a research paper in international conferences before the present GCC conference?**

1. Yes b) No

**Have you ever participated in medical research before in your college, if yes how many?**

1. Yes --------- b) No

**Have you ever published an article in a peer reviewed journal, if yes how many?**

1. Yes -------- b) No

**During your research process, what is the role that your research mentor gave to you (You can select whatever possible)?**

a) Never participated b) Main author c) Co-author d) Data collectors

e) Data entry f) Analysis g) Publication h) Manuscript preparation

i) Involved in all steps of the research

**Is research-funding system provide grants for undergraduate students research at your medical school?**

- - 1. Yes b) No c) don't know

**Personal research experience**

| **Questions** | Yes | No |
| --- | --- | --- |
| a) Have you ever conducted a funded research? |  |  |
| b) Have you ever performed a research that led to positive impact on your society or provided an industrial application? |  |  |
| c) Do you feel confident in interpreting and writing a research paper? |  |  |
| d) Do you think undergraduate students can plan and conduct a research project and write a scientific paper as a part of their curriculum? |  |  |
| e) Do you think medical students can plan and conduct research project without supervision? |  |  |

**Which one of the following motive/s was behind conducting research during medical school?**

| **Questions** | **Agree** | **Neutral** | **Disagree** |
| --- | --- | --- | --- |
| Compulsory research methodology course |  |  |  |
| Facilitating my acceptance to a residency program |  |  |  |
| Positive achievement on my resume |  |  |  |
| Fulfilling research interests |  |  |  |
| Improving my research skills |  |  |  |
| Attaining a research publication |  |  |  |
| Improving health and community care |  |  |  |
| Improve patient's care |  |  |  |

**Which one of the following barrier/s prevents students’ participating in research?**

| **Questions** | **Agree** | **Neutral** | **Disagree** |
| --- | --- | --- | --- |
| Lake of mandatory courses on research methodology |  |  |  |
| Lake of time for research conduction |  |  |  |
| Financial constriction |  |  |  |
| Lake of interest in research |  |  |  |
| Lake of statistical support |  |  |  |
| Lake of (insufficient) mentorship |  |  |  |
| Difficulty in dealing with patients |  |  |  |
| Difficulty in obtaining approval for the study |  |  |  |
