# Supplementary material for: Undergraduate medical research in the Gulf Cooperation Council (GCC) countries: a descriptive study of the students’ perspective
Source: BMC Res Notes. 2018 May 8;11:283. doi: 10.1186/s13104-018-3381-y (PMC5941694; doi:10.1186/s13104-018-3381-y)
Supplement: Supplementary file 2 — Additional file 2: Table S1. Personal experience of participants in research and their believes based on that experience. [file 13104_2018_3381_MOESM2_ESM.docx]

| Personal research experience | n | % |
| --- | --- | --- |
| Ever conducted a funded research | 98 | 43.0 |
| Performed a research that led to positive impact on society or provided an industrial application | 121 | 53.1 |
| Feel confident in interpreting and writing a research paper | 160 | 70.2 |
| Believe that undergraduate students can plan and conduct a research project and write a scientific paper as a part of their curriculum | 199 | 87.3 |
| Believe that Medical students can plan and conduct research project without supervision | 71 | 31.1 |
