# Supplementary material for: Undergraduate medical research in the Gulf Cooperation Council (GCC) countries: a descriptive study of the students’ perspective
Source: BMC Res Notes. 2018 May 8;11:283. doi: 10.1186/s13104-018-3381-y (PMC5941694; doi:10.1186/s13104-018-3381-y)
Supplement: Supplementary file 4 — Additional file 4: Table S2. Academic year-wise comparison of students regarding motives of research. [file 13104_2018_3381_MOESM4_ESM.docx]

| Motivating factors | Academic Year | | | | | | | **P-value** |
| --- | --- | --- | --- | --- | --- | --- | --- | --- |
|  | **1st year** | **2nd year** | **3rd year** | **4th year** | **5th year** | **6th year** | **Intern** |  |
| Compulsory research methodology course | 20.0 | 61.1 | 63.0 | 58.3 | 69.2 | 66.7 | 63.2 | 0.58 |
| Facilitating my acceptance to a residency program | 80.0 | 77.8 | 63.0 | 83.3 | 67.7 | 84.6 | 89.5 | 0.29 |
| Positive achievement on my resume | 60.0 | 66.7 | 78.3 | 72.2 | 80.0 | 87.2 | 94.7 | 0.41 |
| Fulfilling research interests | 60.0 | 66.7 | 71.7 | 75.0 | 70.8 | 87.2 | 78.9 | 0.38 |
| Improving my research skills | 60.0 | 83.3 | 82.6 | 94.4 | 83.1 | 89.7 | 84.2 | 0.18 |
| Attaining a research publication | 80.0 | 77.8 | 82.6 | 72.2 | 81.5 | 76.9 | 78.9 | 0.73 |
| Improving health and community care | 60.0 | 72.2 | 84.8 | 69.4 | 80.0 | 82.1 | 78.9 | 0.53 |
| Improve patient's care | 80.0 | 66.7 | 87.0 | 80.6 | 81.5 | 79.5 | 84.2 | 0.58 |

Results are shown as percentage (%)
