# Supplementary material for: Undergraduate medical research in the Gulf Cooperation Council (GCC) countries: a descriptive study of the students’ perspective
Source: BMC Res Notes. 2018 May 8;11:283. doi: 10.1186/s13104-018-3381-y (PMC5941694; doi:10.1186/s13104-018-3381-y)
Supplement: Supplementary file 4 [file 13104_2018_3381_MOESM3_ESM.docx]

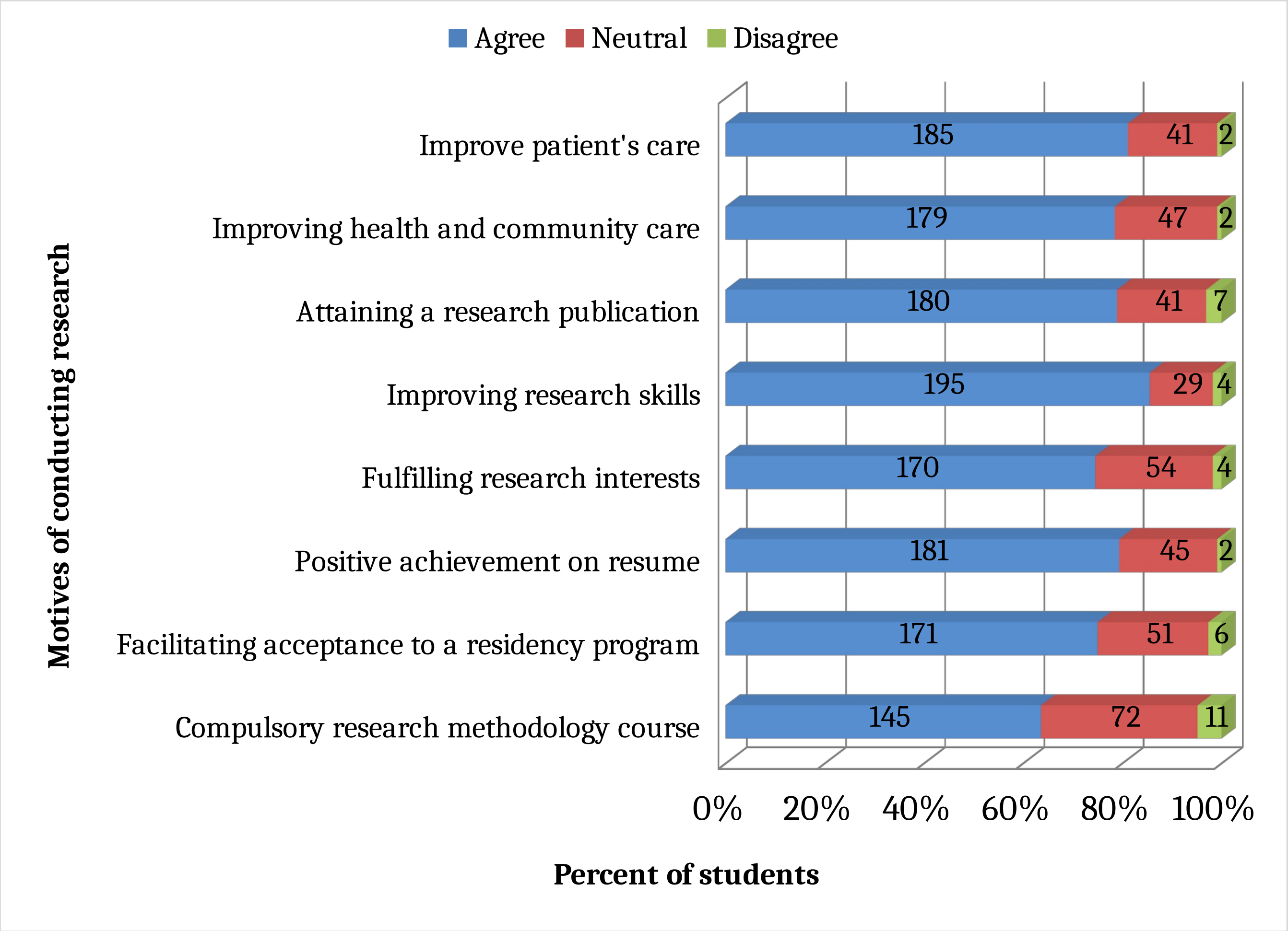
Agree
Neutral
Disagree
Motives of conducting research
Improve patient's care
Improving health and community care
Attaining a research publication
Improving research skills
Fulfilling research interests
Positive achievement on resume
Facilitating acceptance to a residency program
Compulsory research methodology course
185
41
2
179
47
2
180
41
7
195
29
4
170
54
4
181
45
2
171
51
6
145
72
11
0%
20%
40%
60%
80%
100%
Percent of students
